# Evaluation of the ocular microcirculation in brain-dead patients: first step towards a new method of multimodal neuromonitoring?

**DOI:** 10.1186/cc13642

**Published:** 2014-03-17

**Authors:** T Tamosuitis, A Pranskunas, N Balciuniene, V Pilvinis, EC Boerma

**Affiliations:** 1 Lithuanian University of Health Sciences, Kaunas, Lithuania; 2 Medical Centre Leeuwarden, the Netherlands

## Introduction

Multimodal neuromonitoring is a part of goal- directed therapy in severe neurosurgical pathology that leads to better understanding and therefore accurate and timely correction of disturbances of cerebral perfusion. The aim of our study was to evaluate and compare microcirculation in the conjunctiva of the eye and sublingual mucosa in brain-dead patients and healthy volunteers. No studies to our knowledge with this purpose were performed previously. We hypothesized that direct videomicroscopic evaluation of conjunctival microcirculation is linked to cerebral blood flow.

## Methods

We evaluated microcirculation of the eye conjunctiva and sublingual mucosa of 10 brain-dead diagnosed patients and 10 healthy volunteers. Brain-death diagnoses were certified by cerebral angiography. Direct *in vivo *observation of the microcirculation was obtained with sidestream dark-field imaging. Assessment of microcirculatory parameters of convective oxygen transport (microvascular flow index (MFI), proportion of perfused vessels (PPV)), and diffusion distance (perfused vessel density (PVD) and total vessel density (TVD)) was performed according to international criteria.

## Results

All brain-dead patients required vasopressor support to sustain perfusion of donor organs. The MFI of small vessels was significantly lower in brain-dead patients in comparison with healthy controls in ocular conjunctiva (2.6 (2.4 to 2.8) vs. 3.0 (3.0 to 3.0), *P *= 0.03) and in sublingual mucosa (2.8 (2.6 to 2.9) vs. 3.0 (3.0 to 3.0), *P *= 0.04). TVD and PVD of small vessels were significantly lower in brain-dead patients in comparison with healthy controls in ocular conjunctiva (10.2 (5.2 to 14.8) vs. 17.0 (15.4 to 27.2) mm/mm^2^, *P *= 0.023 and 5.2 (2.9 to 7.2) vs. 10.1 (8.5 to 16.7) l/mm, *P *= 0.023), but there was no difference in sublingual mucosa.

## Conclusion

We were able to identify a significant reduction of capillary density in the eye conjunctiva but not in sublingual mucosa when comparing microcirculation of brain-dead diagnosed patients and healthy volunteers. However, the presence of conjunctival flow in case of general cerebral flow is completely absent, making it difficult to use conjunctival flow as a substitute for brain flow.

